# ZFP57 regulation of transposable elements and gene expression within and beyond imprinted domains

**DOI:** 10.1186/s13072-019-0295-4

**Published:** 2019-08-09

**Authors:** Hui Shi, Ruslan Strogantsev, Nozomi Takahashi, Anastasiya Kazachenka, Matthew C. Lorincz, Myriam Hemberger, Anne C. Ferguson-Smith

**Affiliations:** 1 Department of Genetics, University of Cambridge, Cambridge, CB2 3EH UK; 2 Babraham Institute, Epigenetics ISP, Babraham, CB22 3AT UK; 3 Cambridge Centre for Trophoblast Research, University of Cambridge, Cambridge, UK; 4 Present Address: The Francis Crick Institute, 1 Midland Road, London, NW1 1AT UK; 5 Department of Medical Genetics, University of British Columbia, Vancouver, BC V6T 1Z3 Canada

**Keywords:** DNA methylation, KZFPs, ZFP57, Transposable elements, Embryonic stem cells

## Abstract

**Background:**

KRAB zinc finger proteins (KZFPs) represent one of the largest families of DNA-binding proteins in vertebrate genomes and appear to have evolved to silence transposable elements (TEs) including endogenous retroviruses through sequence-specific targeting of repressive chromatin states. ZFP57 is required to maintain the post-fertilization DNA methylation memory of parental origin at genomic imprints. Here we conduct RNA-seq and ChIP-seq analyses in normal and ZFP57 mutant mouse ES cells to understand the relative importance of ZFP57 at imprints, unique and repetitive regions of the genome.

**Results:**

Over 80% of ZFP57 targets are TEs, however, ZFP57 is not essential for their repression. The remaining targets lie within unique imprinted and non-imprinted sequences. Though the loss of ZFP57 influences imprinted genes as expected, the majority of unique gene targets lose H3K9me3 with little effect on DNA methylation and very few exhibit alterations in expression. Comparison of ZFP57 mutants with DNA methyltransferase-deleted ES cells (TKO) identifies a remarkably similar pattern of H3K9me3 loss across the genome. These data define regions where H3K9me3 is secondary to DNA methylation and we propose that ZFP57 is the principal if not sole methylation-sensitive KZFP in mouse ES cells. Finally, we examine dynamics of DNA and H3K9 methylation during pre-implantation development and show that sites bound by ZFP57 in ES cells maintain DNA methylation and H3K9me3 at imprints and at non-imprinted regions on the maternally inherited chromosome throughout preimplantation development.

**Conclusion:**

Our analyses suggest the evolution of a rare DNA methylation-sensitive KZFP that is not essential for repeat silencing, but whose primary function is to maintain DNA methylation and repressive histone marks at germline-derived imprinting control regions.

**Electronic supplementary material:**

The online version of this article (10.1186/s13072-019-0295-4) contains supplementary material, which is available to authorized users.

## Introduction

Kruppel-associated (KRAB) zinc finger proteins (KZFPs) represent one of the largest families of DNA-binding proteins. They are represented in most but not all vertebrate species [1–4], with a recent study mapping the target sites of over 200 KZFPs in human HEK293T cells predominantly to transposable elements including endogenous retroviruses (ERVs) [4]. This is consistent with a small number of more focused studies investigating individual KZFPs in the regulation of transposable elements. For example, KZFPs such as ZFP809 [5], ZFP932 and its paralog Gm15446 [6] have each been shown to repress different retrotransposon families in mouse embryonic stem cells (ES cells). Collectively, these and other studies [7, 8] suggest that the main function of KZFPs is to regulate transposable elements. The binding of KZFPs to retrotransposons is associated with their transcriptional silencing and is mediated by recruitment of the KAP1 (also known as TRIM28 or TIF1β) co-repressor complex, which induces the local acquisition of H3K9me3. Members of the co-repressor complex include the DNA methyltransferases, histone methyltransferase SETDB1 (also known as ESET), heterochromatin protein 1 (HP1), the histone demethylase LSD1, and NuRD histone deacetylase complex [9, 10]. Loss of KAP1 binding leads to the specific loss of H3K9me3 [11, 16].

Individual KZFPs have also been noted to bind to unique regions of the mammalian genome [4, 12–14]. In particular, a role for ZFP57 in the targeted maintenance of genomic imprints has provided novel insights into functions of this family outside the management of repetitive elements. Genomic imprinting is a process that causes genes to be expressed according to their parental origin. Imprinted genes are regulated by parental origin-specific DNA methylation at imprinting control regions (ICRs) that is acquired at different locations in the male and female germlines (germline differentially methylated regions, gDMRs) and maintained after fertilization during preimplantation epigenetic reprogramming [15]. In vivo, ZFP57, with ZFP445, is required to maintain the methylation memory of parental origin during this critical dynamic epigenetic period. ZFP57 binds to all ICRs in ES cells [14, 16–19] with the exception of *Slc38a4*, which appears to be regulated in a different manner [20]. In humans, mutation of ZFP57 has been found in patients with multi-locus imprinting disturbances including transient neonatal diabetes mellitus type 1 (TNDM1) [21, 22]. Furthermore, in mice, ZFP57 exerts a maternal–zygotic effect, whereby deletion of both the maternal gene in oocytes and the zygotic copies in early embryos causes severe loss of methylation imprints at multiple imprinted loci, resulting in embryonic lethality. Homozygous deletion of only the zygotic ZFP57 presents a partially penetrant lethal phenotype [14]. Binding of ZFP57 to other unique regions has also been described, including strain-specific interactions conferred by genotype-specific polymorphisms in ZFP57 recognition sites [17]. Though correlated with strain-specific transcriptional behavior in some instances [17, 23], the functional importance of such interactions at non-imprinted loci in vivo are not known.

To determine the relative importance of ZFP57 binding at different genomic locations, we assessed whether ZFP57 function in ES cells extends beyond the regulation of imprinted regions including at retrotransposons. We found extensive ZFP57 binding at unique germline methylated sites outside imprinted domains, whilst the vast majority of binding was targeted to transposable elements (TEs). Deletion of ZFP57 resulted in loss of KAP1 binding and H3K9me3 at imprinted locations as well as at ~ 100 other unique ZFP57 targets. In contrast to imprinted domains, this was only modestly correlated with changes in DNA methylation and expression of nearby genes suggesting a distinct role, or no role, for ZFP57 at these regions. With few notable exceptions, TEs maintained DNA methylation and H3K9me3 supporting the idea that they may be transcriptionally silenced by multiple redundant mechanisms involving other KZFPs in ES cells [4, 6, 24, 25]. This pattern of retained H3K9me3 was also observed in ES cells derived from embryos with maternal–zygotic ZFP57 deletion. Interestingly, the pattern of ZFP57 binding in ES cells essentially mimicked sites where H3K9me3 was lost in DNA methyltransferase (*Dnmt1/Dnmt3a/Dnmt3b*) triple knock out (DNMT TKO) ES cells [25], indicating a primary role for DNA methylation in the establishment of H3K9me3 via ZFP57. Thus, we propose that the primary role of ZFP57 is in the maintenance of genomic imprints via its DNA methylation-sensitive binding, and that its interaction with other targets occurs by virtue of their existing DNA methylation status, perhaps providing reinforcement of an already silenced state.

### Results

#### Analysis of blastocyst-derived ZFP57 null ES cells

To better understand the role(s) played by ZFP57 during preimplantation development, we have derived ZFP57 null ES cells directly from C57BL/6J (BL6) blastocyst embryos in ground state pluripotency conditions [26]. ZFP57 homozygous mutant null ES cells (ZFP57 KO) were derived from embryos with targeted disruption of coding exon 4 and 5 comprising both the KRAB domain and zinc fingers [18]. Genotyping analysis of ES cells showed the expected Mendelian ratios, enabling selection of both wild-type and mutant male lines for further analysis. In addition, we derived two maternal–zygotic ZFP57 deleted (ZFP57 MZ-KO) ES cell lines on mixed C57BL/6J-129/SvS1 (BL6/129) background. These could not be generated on a pure C57BL/6J background, since homozygous BL6 mutant females do not survive to term. Absence of ZFP57 protein in mutants was confirmed by western blot analysis on whole cell extracts (Additional file 1: Fig. S1a). Quantitative bisulfite pyrosequencing confirmed loss of methylation imprints in all the mutants consistent with previous reports [16, 23], with the exception of KvDMR1, where loss of DNA methylation could only be observed in BL6/129-derived MZ-KO ES cells (Additional file 1: Fig. S1b). Either increased penetrance of the maternal zygotic phenotype or genetic background effects may account for the variable phenotype observed at KvDMR1.

#### Mapping of genome-wide ZFP57-binding sites in ES cells reveals binding events at transposable elements in addition to unique non-imprinted genes

We mapped ZFP57 genomic targets by ChIP-seq in WT and ZFP57 KO ES cell lines using an antibody that recognizes endogenous ZFP57. In total, we identified 1423 high-confidence ZFP57 peaks, where each peak was independently called in at least two of three WT biological replicates. As expected, we observed strong ZFP57 binding at 20 of the 22 known ICR elements, including binding at previously identified tissue specific imprinted genes including *Cdh15* [27], with weaker peaks at the two remaining imprints lying below the peak threshold (*Gpr1* and *Mcts2*). As in our previous report, no binding was detected at the *Slc38a4* DMR [17], in agreement with a study demonstrating a role for H3K27me3 in regulating its germline imprinting [20].

**Fig. 1:**
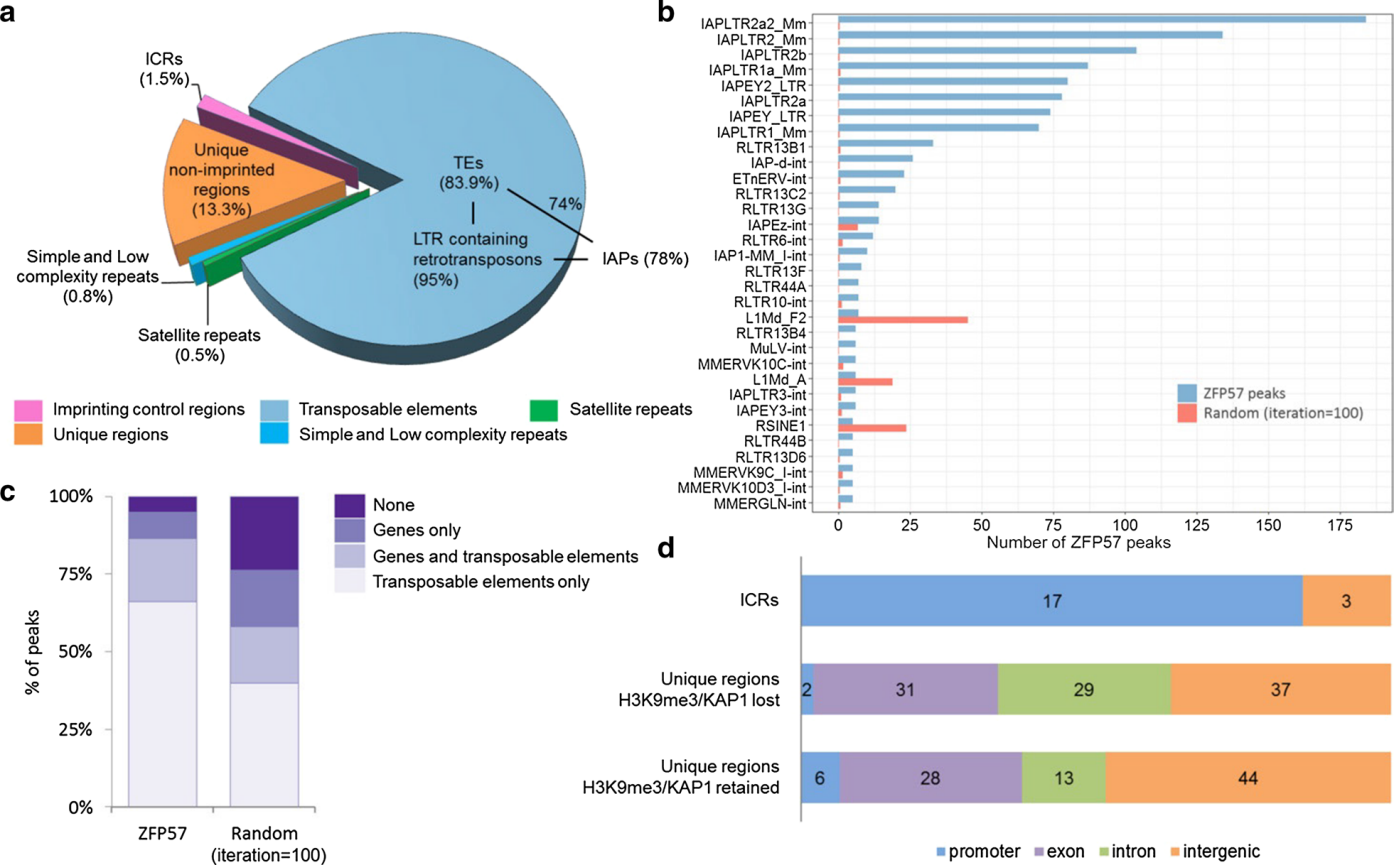
Genome-wide mapping of ZFP57 binding sites in ES cells. **a** Distribution of ZFP57 binding sites with respect to unique regions and repetitive elements. **b** Top 32 TE classes bound by ZFP57, ranked by number of ZFP57 peaks. IAPLTR2a2_Mm represents the most commonly targeted TE by ZFP57. **c** Significant enrichment of ZFP57 peaks at TEs relative to random peak permutations, number of iterations = 100, binomial *P* < 0.0001. **d** Non-repeat-associated peaks bind distinct genomic regions within ICRs and non-imprinted regions

Using this stringent cutoff, the remainder of the ZFP57 peaks was further characterized according to their relative location at unique regions and at different types of repetitive elements in the mouse genome. We found 13.3% (*n* = 189) of targets mapped to non-repetitive regions, a further small subset of peaks 1.3% (*n* = 19) at non-transposon derived repeats (e.g., simple, low complexity and satellite repeats), whilst the vast majority of the peaks 83.9% (*n* = 1194) targeted transposable elements (Fig. 1a). Furthermore, ZFP57 is specifically enriched at LTR-containing retrotransposons (*n* = 1061) of which the majority (*n* = 795) were IAPs, whilst LINEs and SINEs were depleted amongst ZFP57-bound elements (Fig. 1b). TE enrichment in the ZFP57-bound subset of repeats was highly significant in comparison to the rest of genome (Fig. 1c, *P *< 0.0001). In total, ~ 7–8% of all IAPs present in the mouse genome are bound by ZFP57 in ES cells. This binding to repeats was specific, since only uniquely aligned reads were used for peak calling (based on paired-end read mapping) (Additional file 1: Fig. S2a). Since TE enrichment analysis using only unique mapped reads may introduce bias toward TEs with better mapping ability, we repeated the peak calling incorporating multimapped reads using random repeat assignment. Of the 523 additional peaks called in this way, 487 again mapped to IAP elements confirming ZFP57 enrichment at these LTR retrotransposons (Additional file 1: Fig. S2b).

Analysis of non-repeat-associated ZFP57 peaks revealed a distinct distribution of imprinted versus non-imprint bound sites with respect to gene features. Whilst maternally methylated ICRs coincide with gene promoters, the majority of non-imprint associated peaks was equally distributed within gene exons, introns and intergenic regions (Fig. 1d). Indeed, very few promoter-bound sites lay outside imprinted regions, but notably included the promoter of the *Dux* gene—a transcription factor involved in zygotic genome activation at the two-cell stage [28, 29]. Our analysis of data from Imbeault et al. revealed that the human DUX4 promoter can be targeted by multiple KZFPs including ZFP57 [4], consistent with our observation that H3K9me3 is only partially lost at this locus in ZFP57 mutants.

#### Distinct H3K9me3- and KAP1-binding profiles at ZFP57-bound unique regions and transposable elements

**Fig. 2:**
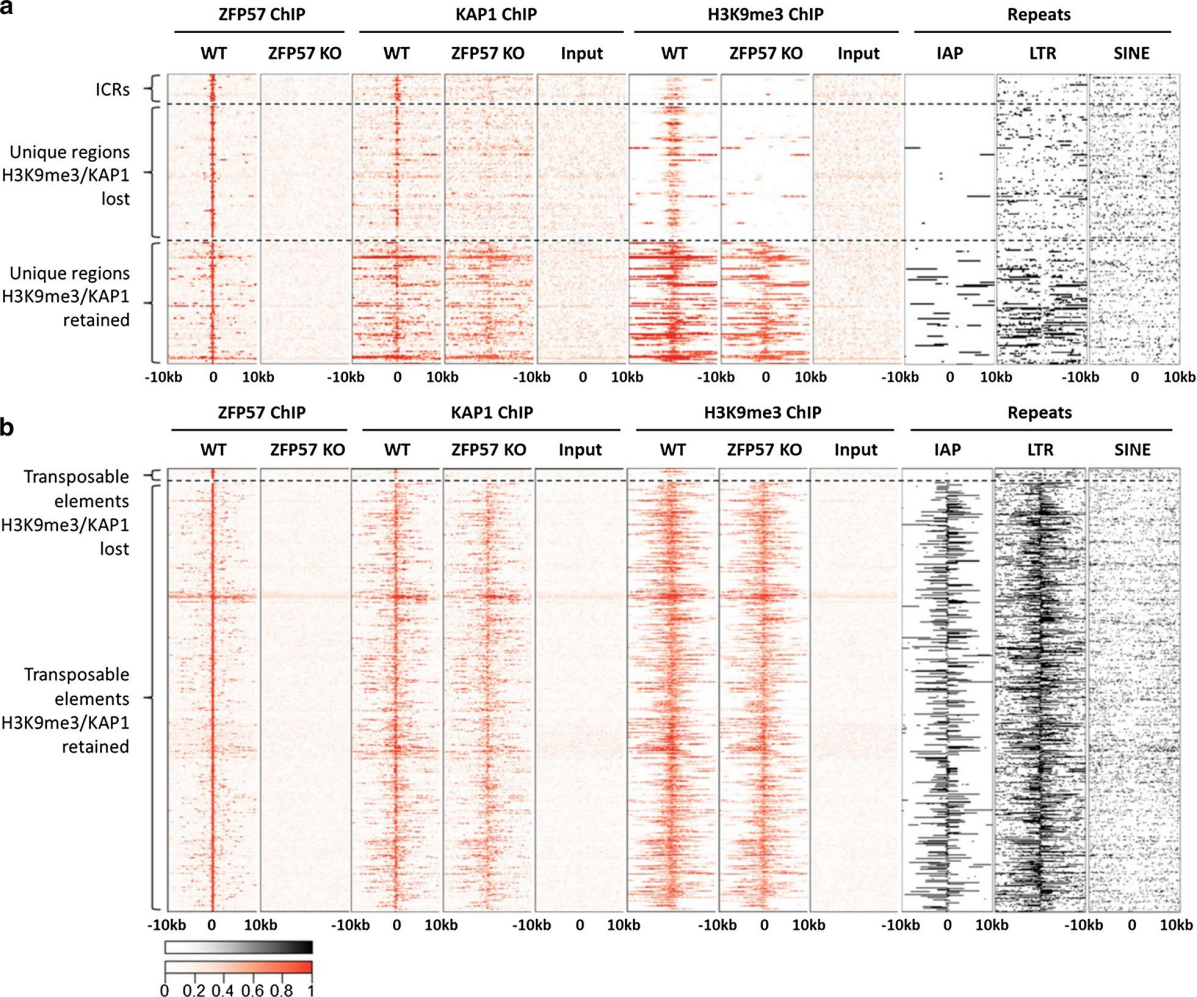
Distinct H3K9me3 and KAP1 binding profile at ZFP57-bound unique regions and transposable elements. **a** Heat maps of ChIP-seq enrichment signal (red) within ICRs/unique regions and **b** transposable element-associated binding sites. Enrichment of IAP, LTR and SINE elements is shown as black-and-white heat maps. Within unique regions, significant enrichment for LTRs nearby the H3K9me3 retaining group compared to the H3K9me3 losing group was observed by calculating distance to the nearest LTR from each peak (unpaired two-tailed *t* test, *P* < 0.001)

KZFPs are associated with the KAP1-mediated recruitment of SETDB1 and in turn H3K9me3 deposition. We therefore examined the presence or absence of KAP1 and H3K9me3 in ZFP57 KO cells by ChIP-seq. Once again, using ICRs as internal controls, we found the expected loss of KAP1 binding at almost all ICRs (*n* = 20), of which 17 also exhibited complete loss of H3K9me3 (Fig. 2 and Additional file 1: Fig. S3). Interestingly, H3K9me3 was also lost at the KvDMR1 ICR—the only DMR retaining DNA methylation in BL6 zygotic KO ES cells (Additional file 1: Figs. S1b, S3). Three ICRs: *Grb10*, *Fkbp6* and *Peg10* exhibited varying degrees of loss of H3K9me3 and KAP1 binding (Additional file 1: Fig. S4).

We next analyzed the genome-wide distribution of KAP1 and H3K9me3 at different categories of ZFP57 peaks in WT and KO cells. Approximately, one half (*n* = 98, 52%) of the unique non-ICR peaks exhibited complete loss of KAP1 and H3K9me3 in ZFP57 mutant ES cells, whilst the rest (*n* = 91, 48%) retained KAP1 binding and H3K9me3 (Fig. 2a). Upon further inspection of the H3K9me3-retaining group, it was revealed that although mapping to unique sequences, they are found in more repeat-dense genomic regions with specific enrichment of LTR-containing retrotransposons (*P* < 0.001, unpaired two-tailed *t* test) encompassing IAPs (Fig. 2a, right panel). We speculate that KAP1 binding (presumably recruited by other KZFPs) at those nearby retrotransposons maintains a generally repressed state of the entire region irrespective of ZFP57 binding.

In contrast to the unique ZFP57 target sites, virtually all ZFP57 peaks at transposable elements (98%; *n* = 1168) retained H3K9me3 levels (Fig. 2b). Only 26 (2.2%) TEs showed loss of KAP1 and H3K9me3 and these were mainly SINEs, non-IAP retroelements or solo IAP-derived LTRs. We thus conclude that despite widespread binding of ZFP57 to transposable elements, only a very small subset of them is dependent on this to maintain H3K9me3.

Finally, we mapped H3K9me3 profiles in ES cells derived from maternal–zygotic deletion of ZFP57 (Additional file 1: Fig. S5). Despite the more penetrant phenotype in vivo of the maternal zygotic deletion [14], we observed a nearly identical pattern of H3K9me3 loss/retention over the unique and repeat-associated subsets of peaks in ZFP57 MZ-KO ES cells. These results show for the first time, that the epigenetic profile of H3K9me3 repressive marks is largely equivalent in both zygotic and maternal–zygotic ZFP57 mutant ES cells.

#### Transcriptional profiling identifies few specific non-imprinted ZFP57 target genes

To determine the functional consequences of ZFP57 deletion on the transcriptome, total RNA sequencing post-ribosomal RNA (rRNA) depletion was performed on these same control and mutant cell lines.

**Fig. 3:**
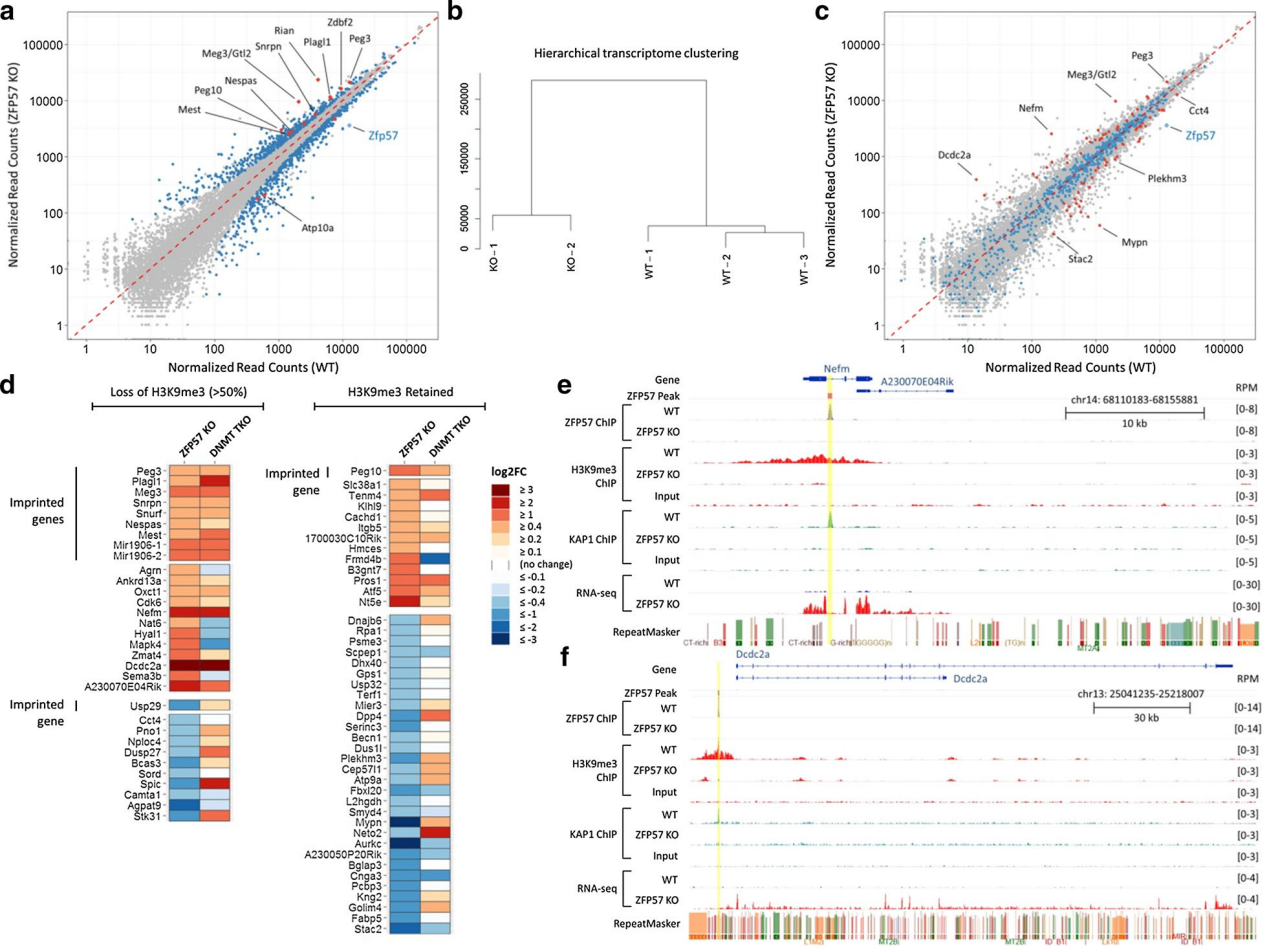
ZFP57 KO ES cells show perturbed expression of imprinted and non-imprinted genes. **a** Scatterplot of gene expression in WT and ZFP57 KO ES cells with differentially expressed genes shown in blue, of which the known imprinted genes are highlighted in red. **b** Hierarchical transcriptome clustering analysis showing clear segregation of WT versus KO cells. **c** Scatterplot as in **a** but highlighted all the genes < 20 kb of ZFP57 peak, blue = no significant change in expression of associated genes (*n* = 673), red = differentially expressed (*n* = 75). **d** Gene expression differences within the 75 genes, with ZFP57 peaks losing H3K9me3 shown on the left, and those retaining H3K9me3 shown on the right. Expression of ZFP57 KO and DNMT TKO versus their corresponding WT control is shown side by side for comparison. **e** Genome browser screenshots for the two highest upregulated non-imprinted genes Nefm and **f** Dcdc2a, showing relative location of ZFP57 peak, H3K9me3/KAP1 enrichment and RNA-seq tracks for each gene

Genome-wide differential expression analysis revealed statistically significant altered expression of 1080 genes in ZFP57 KO cells in comparison to WT, which included many known imprinted genes in agreement with previously demonstrated roles of ZFP57 in imprint regulation (Fig. 3a and Additional file 2: Table S1). Quantification of all annotated gene transcripts followed by hierarchical clustering analysis indicated clear segregation of the WT and mutant cell lines (Fig. 3b). To determine whether non-imprint bound ZFP57 regions might regulate expression of nearby genes, we analyzed expression of all genes in close proximity (< 20 kb) to ZFP57 peaks (*n* = 748, Fig. 3c). We found that only a small subset (*n* = 75) of differentially expressed genes harbored ZFP57-binding sites or have ZFP57 peaks located nearby (< 20 kb). Thus, the majority of differentially expressed genes may not be direct targets of ZFP57 and instead may represent secondary effects of a perturbed imprinted gene network.

We ascertained whether the 75 ZFP57-bound differentially expressed genes were misregulated as a direct consequence of loss of H3K9me3 and/or DNA methylation in ZFP57 KO ES cells by comparing this with their expression levels in DNMT TKO ES cells lacking both maintenance and de novo DNA methyl transferases [25]. These cells are devoid of any detectable 5-methylcytosine (5mC) in the genome, and as ZFP57 binding is DNA methylation dependent, they are unable to target ZFP57 binding. We found that imprinted genes upregulated in ZFP57 KO ES cells had a strong tendency to be also upregulated in DNMT TKO cells suggesting these are direct ZFP57 targets (Fig. 3d, top). In addition, several non-imprinted genes were also coordinately upregulated in both mutants, whilst no such correlation could be seen in genes that are downregulated in ZFP57 null ES cells (Fig. 3d, bottom) indicating that those are likely to be secondary effects. Amongst the strongest upregulated non-imprinted genes in both mutants were the *Nefm* and *Dcdc2a* genes (Fig. 3e, f). In both cases, loss of ZFP57 led to loss of KAP1 binding, and abolition of a repressive H3K9me3 domain around the gene indicating a primary role for DNA methylation in the recruitment of repressive epigenetic states at these regions via ZFP57.

In conclusion, we found a large network of gene deregulation occurring in ZFP57 KO ES cells, however, apart from imprints, only a small proportion of non-imprinted genes are likely to be directly regulated by ZFP57.

#### ZFP57 is not required for transposable element silencing in ES cells

**Fig. 4:**
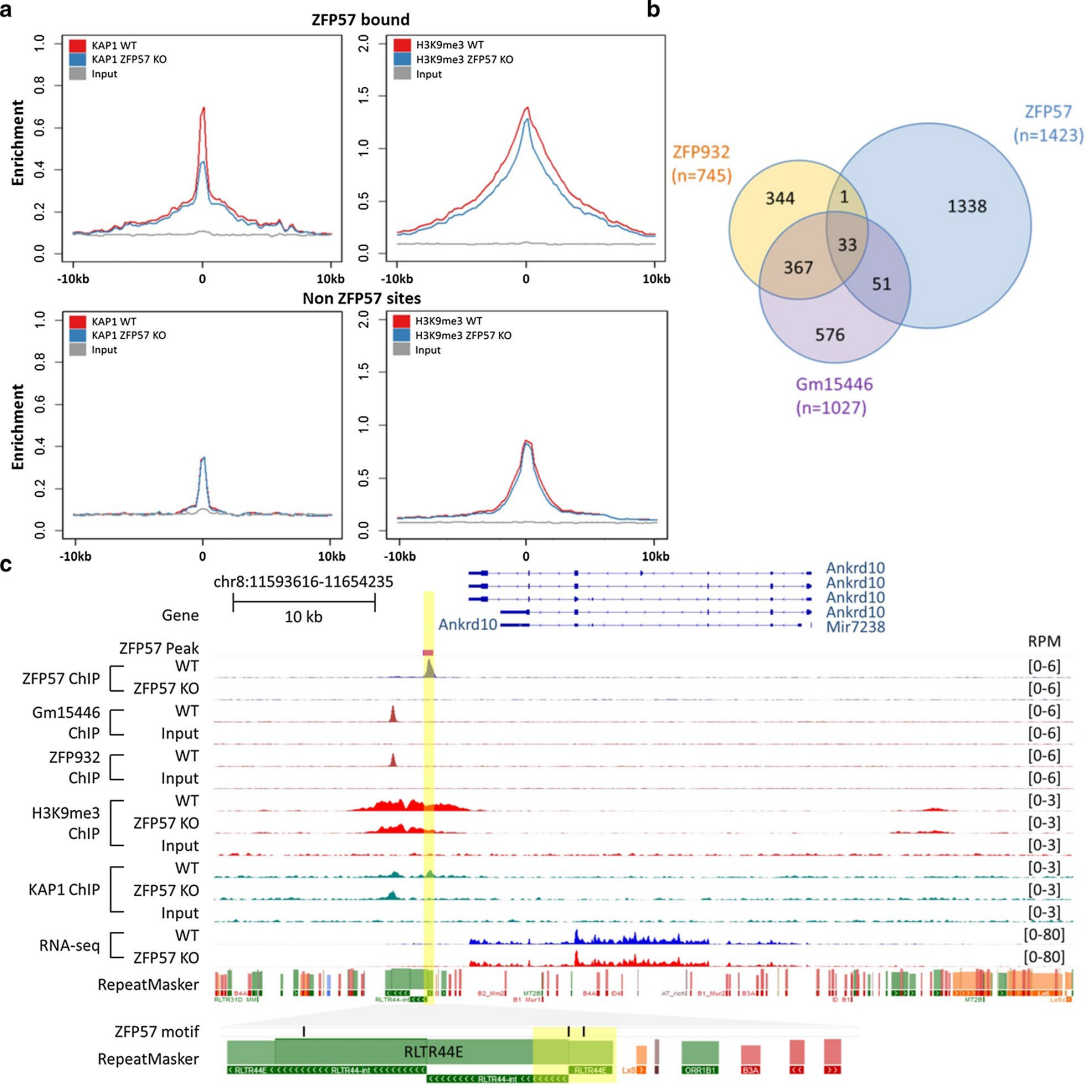
ZFP57 plays a largely redundant role in transposon silencing. **a** Reduction of KAP1 ChIP signals in ZFP57 KO under TE-bound ZFP57 peaks, which leads to very modest reduction in H3K9me3 levels (top), no such reduction was observed at other KAP-bound TEs in absence of ZFP57 binding (bottom). **b** Venn diagram showing partial overlapping ZFP57, ZFP932 and Gm15446 peaks. 33 ERVs were found to be triply co-bound. **c** Genome browser screenshot of a triply co-bound RLTR44E retroelement near the Ankrd10 gene. Notably, there is a partial loss of H3K9me3 under the ZFP57 peak in ZFP57 KO but not adjacent ZFP932/Gm15446 peaks, thus maintaining overall silencing of the repeat and unchanged levels of the proximal gene

Given the widely accepted role of KZFPs in silencing retroelements, we further explored whether ZFP57 binding to transposable elements is functionally relevant or perhaps a secondary effect of DNA methylation at these loci. The vast majority of ZFP57-bound transposable elements retained H3K9me3 in mutants (Fig. 2b) and remained transcriptionally repressed (Additional file 1: Fig. S6 and Additional file 2: Table S2). Interestingly, despite the overall retention of H3K9me3 enrichment, we observed a reduction of KAP1 signal at ZFP57-bound TEs in the mutants (Fig. 4a), indicating that ZFP57 at least in part contributes to KAP1 recruitment. We hypothesized that the retained repression of retrotransposons in ZFP57 KO ES cells might be mediated by binding of other KZFPs recruiting KAP1 at levels sufficient to maintain H3K9me3. A recent screen for KZFPs that target retrotransposon silencing identified ZFP932 and Gm15446 as partially redundant factors binding to endogenous retroviruses including IAPs [6]. We overlapped these published binding sites with those at ZFP57 and identified a subset (*n* = 85) of targeted transposable elements that are jointly bound by ZFP57 (Fig. 4b). One example of this is shown for the RLTR44E retroelement located ~ 2 kb downstream from the *Ankrd10* gene and harbors adjacently located ZFP57 and ZFP932/Gm15446 peaks (Fig. 4c). In ZFP57 null ES cells, KAP1 is specifically lost under the ZFP57 peak, whilst being maintained at the neighboring site of two other KZFPs. Consequently, the region of the repeat maintains overall H3K9me3 levels and neither reactivation of RLTR44E nor *Ankrd10* was observed in ZFP57 mutants. Amongst triply bound transposable elements in general, no reactivation was observed in either the ZFP932/Gm15446 double deletion or ZFP57 KO ES cells (with the exception of the IAP element in the vicinity of *Bglap3*), indicating potential for redundant functions of KZFPs (Additional file 1: Fig. S7).

Intriguingly, ~ 2% of transposable elements (*n* = 26) bound by ZFP57 did exhibit loss of KAP1 and H3K9me3 (Fig. 2b). However, apart from two notable exceptions, this was not associated with transcriptional activation of the repeat itself or of the nearby coding gene. In one case, loss of ZFP57 binding at the intergenic MER20 (a DNA transposon) was associated with an increase in the expression of an unannotated transcript comprising both unique regions and a cluster of transposable elements (Additional file 1: Fig. S8). In the other example, the IAP-derived LTR showed loss of H3K9me3 and KAP1 binding and this was associated with increased expression of the host coding gene *Mapk4* (Additional file 1: Fig. S9) [25]. This element comprised the LTR only and not the full length IAP, supporting the general rule that IAPs are not affected by ZFP57 ablation.

Together, our data suggest that ZFP57 is not required for repression of transposable elements to which it binds. This might be partially explained by other KZFPs binding alongside ZFP57 to recruit repressive states to these elements. However, the highly redundant and combinatorial nature of KZFPs binding to transposable elements makes it difficult to ascertain the complexities of these relationships.

#### Relationship between DNA methylation and H3K9me3 at ZFP57 targets

**Fig. 5:**
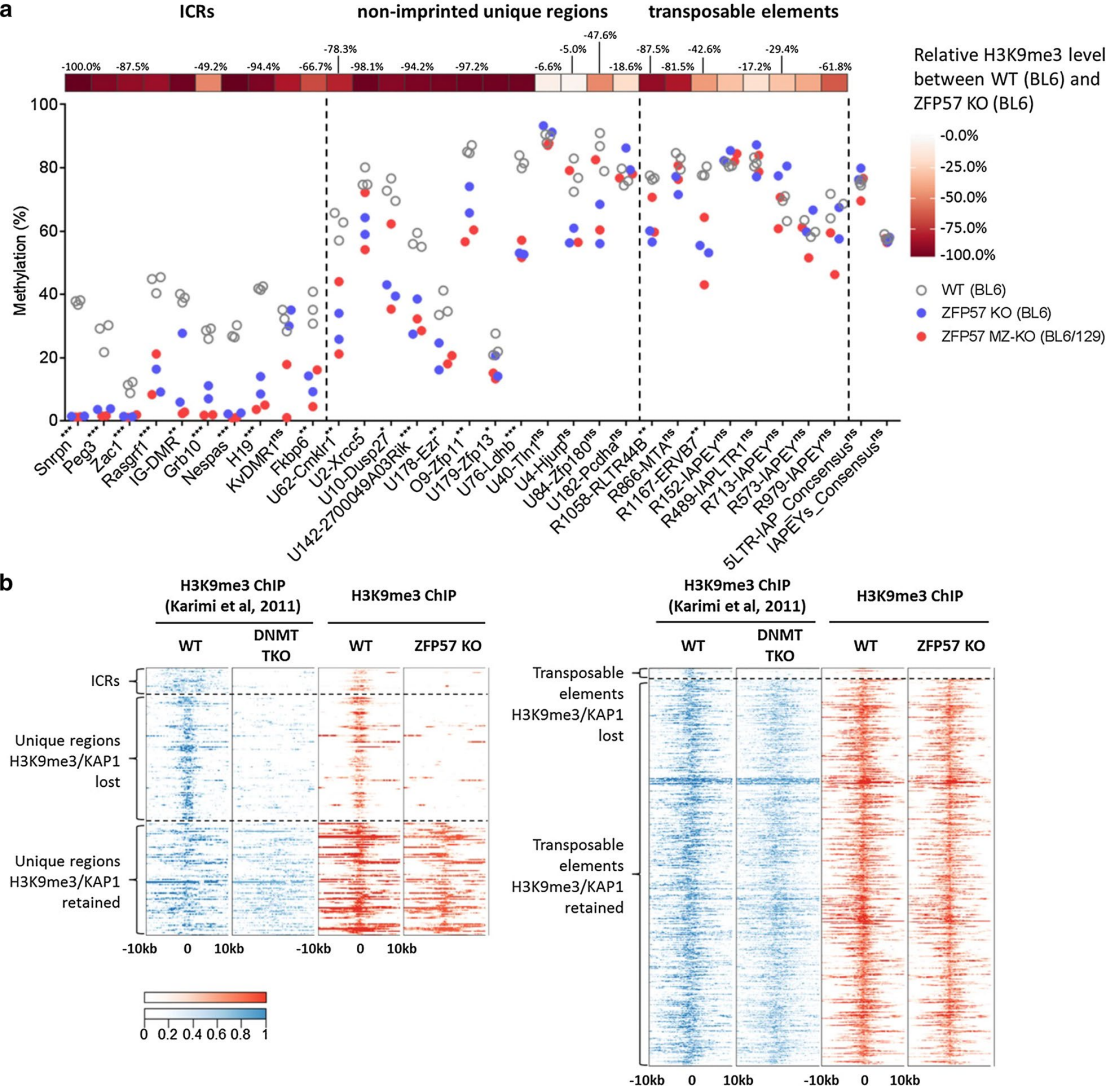
Relationship between DNA methylation and H3K9me3 at ZFP57-binding sites in ES cells. **a** Relationship between loss of DNA methylation and loss of H3K9me3 at select ICRs, unique regions and transposable elements bound by ZFP57. 5′ LTR-IAP and IAPEY consensus regions were used as control. ns, not significant, *P < 0.05, **P < 0.01, ***P < 0.001; unpaired two-tailed *t* test. **b** Heat maps of ChIP signals for H3K9me3 in DNMT TKO (blue trace) and ZFP57 KO (red trace) ES cells at ZFP57-bound unique regions (left) and transposable elements (right)

To explore the relationship between DNA methylation and H3K9me3 at different types of ZFP57 targets in more detail, we analyzed DNA methylation levels at these regions using quantitative bisulfite pyrosequencing. As expected, we found that loss of ZFP57 and H3K9me3 was associated with loss of DNA methylation at imprints. Regions retaining H3K9me3 (both transposons and unique non-imprinted peaks) predominantly retain their DNA methylation status (Fig. 5a). Interestingly, non-imprinted regions that lost H3K9me3 retained considerable levels of DNA methylation indicating that DNA methylation at these loci is not dependent on H3K9me3.

Conversely, to test if DNA methylation is necessary to confer H3K9me3 at these regions, we compared the profile of H3K9me3 in ZFP57 KO cells with that of DNA methylation-deficient (DNMT TKO) cells [25]. Strikingly, our analysis identified the pattern of H3K9me3 to be virtually identical between the TKO and ZFP57 mutants: the repressive mark was lost at ICRs and at the exact same subset of non-imprinted unique regions, whilst it was maintained over TE-associated ZFP57 peaks (Fig. 5b). These data suggest two types of relationships between H3K9me3 and DNA methylation mediated by ZFP57: imprinted and non-imprinted unique regions where H3K9me3 is dependent on DNA methylation and TEs, where H3K9me3 can be maintained in the absence of DNA methylation. Importantly, in both instances, ZFP57 recruits KAP1.

Finally, we queried the global extent to which ZFP57-binding sites can explain DNA methylation-dependent H3K9me3 in DNMT TKO cells. In total, 15.6% (*n* = 91) of all H3K9me3 sites that were lost in the TKO mutant had both a strong ZFP57 and KAP1 enrichment signal (Additional file 1: Fig. S10). Curiously, the remaining H3K9me3-depleted regions (*n* = 492) were not associated with KAP1 indicating that ZFP57 is the main, if not sole, KZFP that recruits repressive histone marks in a DNA methylation-dependent manner in ES cells. This also indicates that KAP1-independent processes regulate H3K9me3 at the other sites.

#### ZFP57 maintains germline-derived DNA and H3K9 methylation at both imprinted and non-imprinted regions during preimplantation development

Imprinting control regions acquire differential DNA methylation in gametes, which is then selectively maintained through preimplantation stages of development. This maintenance has been shown to be fully ZFP57 dependent in ES cells and for a subset of ICRs in vivo [14, 18]. We investigated whether non-imprinted unique ZFP57 targets in ES cells are also germline methylated and whether they too are protected from post-fertilization genome-wide erasure of DNA methylation.

**Fig. 6:**
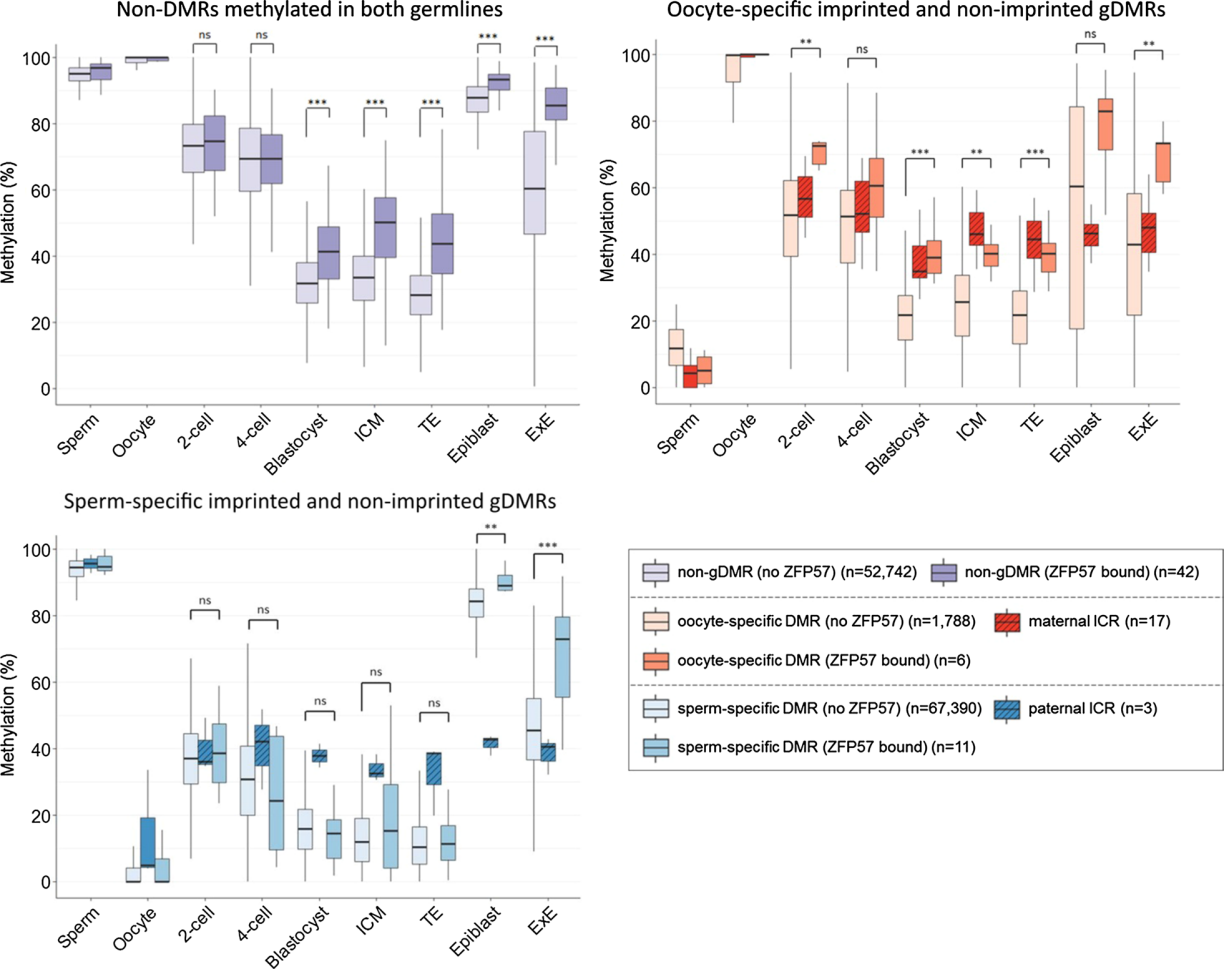
ZFP57 maintains DNA methylation at both imprinted and non-imprinted regions during preimplantation development. DNA methylation levels of non-DMRs, oocyte and sperm-specific germline DMRs bound by ZFP57 (darker colored) versus those not bound by ZFP57 (light colored) during preimplantation development and in E6.5 epiblast and extraembryonic ectoderm [30–32]. Known ICRs are shown as hatched boxes for comparison. ICM, inner cell mass; TE, trophectoderm; ExE, extraembryonic ectoderm; ns, not significant, *P < 0.05, **P < 0.01, ***P < 0.001; Mann–Whitney *U* test

Analysis of publicly available datasets [30–32] revealed that indeed the majority of unique non-imprinted targets of ZFP57 where methylation data are available are methylated in both germlines (*n* = 42), with a few that are either oocyte (*n* = 6) or sperm (*n* = 11) specific gDMRs. We analyzed the DNA methylation dynamics at these regions throughout pre-implantation development and found that ZFP57 binding conferred significant protection against post-fertilization demethylation at oocyte-specific gDMRs as well as non-DMR regions methylated in both germ cells (Fig. 6 and Additional file 2: Table S3). Most intriguingly, allele-specific methylation analysis revealed preferential protection of the maternal allele resulting in formation of transient DMRs post-fertilization (Additional file 2: Table S4). In contrast to known imprinted ICRs, these transient oocyte-specific gDMRs get resolved by acquiring DNA methylation in the post-implantation epiblast, resembling patterns that have been previously reported for placenta-specific imprinted and transient maternal gDMRs [27, 32–34]. Sperm-specific methylated regions, with exception of the three known paternal germline imprints, are not generally protected from DNA methylation reprogramming despite being ZFP57 bound in our ChIP-seq ES cell data.

We next analyzed H3K9me3 level dynamics at these regions using recently published datasets [35]. Known maternally methylated ICRs were found to be enriched for H3K9me3 in the MII oocyte and retained this state in accordance with their DNA methylation. Furthermore, non-imprinted transient gDMR and non-DMR regions that are both DNA methylated and bound by ZFP57 had significantly higher H3K9me3 levels in the oocyte than those without ZFP57. This was also true at post-fertilization stages (Additional file 1: Figure S11). In contrast, sperm chromatin is nucleosome depleted and hence is generally devoid of H3K9me3 modification regardless of gDMR or ZFP57 binding status [35, 36].

Taken together, we conclude that ZFP57 preferentially protects maternal methylation at bound regions against post-fertilization epigenetic reprogramming, whilst playing no role in protecting non-imprinted paternally inherited methylation.

## Discussion

In the present study, we demonstrate that, in ES cells, ZFP57 binds not only to ICRs, but also to non-imprinted unique regions and transposable elements, in particular, to IAPs. We have performed a detailed epigenetic and transcriptomic analysis of ZFP57 mutant ES cells allowing us to ascertain the relative functional roles played by ZFP57 within different genomic locations. We have determined the hierarchical relationship between DNA methylation and H3K9me3 at ZFP57-bound regions, and assessed whether ZFP57 maintains DNA methylation at non-imprinted loci during post-fertilization epigenetic reprogramming.

We have identified a subset of unique non-imprinted genomic regions, which depend on DNA methylation and ZFP57 binding for the recruitment of KAP1 and H3K9me3. Only a small proportion of these is associated with significant changes in gene expression in ZFP57 mutants. A striking difference between ICRs and non-imprinted unique ZFP57-bound regions is that the former are predominantly found at promoters, but the latter bind exonic, intronic and intergenic regions. A subset of ZFP57 peaks map to the 3′ exons of several protein coding genes (*n* = 16) including other Zn-finger transcription factors such as ZFP13, ZFP180 and ZFP629. These genes are expressed in WT ES cells and their expression remains unchanged in mutant ES cells despite loss of KAP1 and H3K9me3 over the 3′ end of these genes. Consistent with this, it has been postulated that 3′ end H3K9me3 recruitment may promote genomic stability and prevent recombination between homologous Zn-finger proteins rather than directly regulating their expression [37, 38].

DNA methylation and H3K9me3 generally co-localize at repressed genomic regions. However, it is not clear where DNA methylation is the driver for the recruitment of H3K9me3 or where it might occur as a secondary consequence of H3K9me3. Studies to investigate this ‘cause or consequence’ relationship are confounded by the lethality of cells harboring mutations in the H3K9me3 machinery [11, 39–41]. Karimi et al. have suggested that DNA methylation and H3K9me3 serve to repress distinct sets of genes as well as some classes of retroelements and showed that only a small subset of regions lose their H3K9me3 in DNA methylation-deficient (DNMT TKO) ES cells [25]. Comparison of the pattern of H3K9me3 loss in DNMT TKO cells with that in ZFP57 KO cells revealed remarkable similarity. The comparison of H3K9me3 in DNMT TKO and ZFP57 KO has therefore identified regions where the presence of H3K9me3 is dependent on DNA methylation and determined that it is ZFP57 bound to methylated DNA that is responsible for the H3K9me3 recruitment.

Recently, we showed that another KZFP member, ZFP445 also binds the methylated allele at imprinted gDMRs and contributes to maintenance of DNA methylation at a subset of ICRs along with ZFP57 in vivo [19]. However, data presented here suggest that at least in context of mouse embryonic stem cells, ZFP57 is both necessary and sufficient to recruit KAP1 and maintain H3K9me3/DNA methylation at imprints. Furthermore, our analysis of ZFP445 binding sites (data not shown) revealed very little overlap with ZFP57 outside of imprinted regions with TE binding being a unique feature of ZFP57. This is in agreement with our finding that ZFP57 binding is responsible for almost all of the KAP1-bound fraction DNA methylation-dependent H3K9me3-modified regions found in DNMT TKO ES cells. Thus, we conclude that ZFP57 is the major KZFP that binds DNA in a methylcytosine-dependent manner in mouse ES cells.

While complete loss of KAP1 and H3K9me3 was observed at ICRs and over half of ZFP57 peaks at unique non-imprinted regions, we found only ~ 2% of repeat-associated ZFP57 peaks exhibited complete loss of these marks. Our data support the hypothesis that in mammalian ES cells, retroviral silencing is mediated by multiple factors acting in a redundant manner. Indeed, we identified extensive overlaps with two other KZFPs (ZFP932 and Gm15446) previously demonstrated to recruit KAP1/H3K9me3 repressive marks to ERVs including IAPs [6]. In this context, it is interesting to consider the DNA methylation-sensitive nature of ZFP57 binding [18, 42]. This property makes it an unlikely candidate KZFP for the initiation of silencing at the integration site of ERV retro-transposons, since it would require DNA methylation to be established first in order for binding to occur. Rather, it may contribute to silencing initiated by other KZFPs such as ZFP809 binding to invariable PBS regions of IAPs [5, 8]. Recent studies have suggested that LTR regions have the potential to function as enhancer and/or alternative promoter elements and can be co-opted by the host genome in regulation of its own gene expression [43–46]. The ability of a KZFP to recruit repressive chromatin states in a methylation-sensitive manner could provide an additional layer of modulation at such loci. However, we also cannot rule out the possibility that ZFP57 binding at these elements is a functionally irrelevant secondary consequence of DNA methylation occurring at already H3K9me3-repressed LTRs that contain the rather common ZFP57 hexanucleotide binding motif. In the latter case, IAPs may even act as a sink and/or buffer for excess ZFP57 protein.

We can now speculate about the function and evolution of ZFP57 in the genome. Indeed, we have shown that ZFP57 does not affect silencing of endogenous retroviral sequences but rather is required for retention of DNA methylation and H3K9me3 at the imprinted ICR regions as well as unique non-imprinted regions. Most strikingly, in cases where these non-imprinted regions are methylated in both germ lines, ZFP57 binding is associated with preferential protection of the maternally inherited regions resulting in DMR formation post-fertilization. This may explain the much higher prevalence of maternally methylated ICRs and highlights the unique characteristics of the three paternal imprints.

The function, if any, of non-imprinted unique ZFP57 bound regions remains to be determined. Although many of these are methylated in oocyte and present a DMR at the blastocyst stage, they typically acquire biallelic methylation later in the post-implantation epiblast. Furthermore, our transcriptomic analysis suggests that apart from known imprinted genes, very few genes become deregulated as a direct consequence of ZFP57 loss. We therefore propose that ZFP57 evolved from ancestral KZFPs originally acting to manage retrotransposons, but now functions to control genomic imprinting and not to regulate retrotransposons. This is consistent with the finding that ZFP57 also contributes to the regulation of imprinting in humans [22], a species that lacks the CpG-rich IAP retrotransposon sequences to which ZFP57 binds in the mouse.

## Additional files

**Additional file 1: Figure S1.** Analysis of blastocyst-derived ZFP57 null ES cells. **a** Western Blot analysis confirming absence of detectable ZFP57 protein. **b,** Bisulfite pyrosequencing of ICRs in WT, ZFP57 KO and ZFP57 MZ-KO ES cells. **Figure S2.** IGB viewer snapshot showing representative ZFP57 peak at IAPLTR2a2_Mm retroelements, which harbors the most number of ZFP57 peaks. Paired-end sequencing, together with sequence divergence of the retroelements allowing us to map ChIP-seq reads to individual transposable elements in the genome. The color scale indicates the mapping quality score (MAPQ) for each read pair. MAPQ = $- 10log_{10} P$ where *P* is the probability that true alignment belongs elsewhere. **b,** Transposable elements indicated by alignments of “multi-mapping reads. ZFP57 targets of these elements show KAP1 binding and H3K9me3 enrichment in both WT and KO cells, and no reactivation has been observed (data not shown). **Figure S3.** Genome browser screenshots for *Snrpn*, *H19* and *KvDMR1* imprinted loci. **Figure S4.** Genome browser screenshots for *Grb10*, *Fkbp6* and *Peg10* imprinted loci. **Figure S5.** Heat maps showing signal enrichment of H3K9me3 at ZFP57 binding sites in ZFP57 KO and MZ-KO ES cells. **Figure S6.** Transposable elements bound by ZFP57 are not reactivated in ZFP57 KO ES cells. **a,** Expression status of ZFP57 bound transposable elements in WT and ZFP57 mutants. **b,** Scatterplot showing no transcriptional changes of TEs (FPKM ≥ 0.1 in WT or KO) in ZFP57 KO cells versus WT. **Figure S7.** Analysis of expression of transposable elements jointly bound by ZFP57, ZFP932 and/or Gm15446 in different mutant ES cells. Top left – ZFP57 KO, Top right – ZFP932/Gm15446 DKO, Bottom left – KAP1 KO. The IAP retroelement in vicinity of Bglap3 locus is not shown in the ZFP932/Gm15446 DKO as its expression far exceeds other jointly bound TEs [6]. **Figure S8.** Genome browser screenshot showing loss of ZFP57 binding at MER20 DNA transposon resulting in loss of KAP1 and H3K9me3 and upregulation of an unannotated transcript. **Figure S9.** Genome browser screenshot showing loss of KAP1 and H3K9me3 in solo LTRs located within and adjacent to Mapk4 gene, and an increase in Mapk4 levels in ZFP57 KO ES cells. **Figure S10.** Heat maps of DNA methylation-dependent H3K9me3 enriched regions in WT and DNMT TKO cells (blue colored heat map) [25]. Enrichment of ZFP57, KAP1 and H3K9me3 in ZFP57 KO ES cells (red) at the same regions. **Figure S11.** ZFP57 maintains H3K9me3 methylation during preimplantation development. H3K9me3 methylation level dynamics during preimplantation development at non-DMR regions methylated in both germ lineages (left) and oocyte-specific germline DMRs (right). Light colored boxes show non-ZFP57 bound (*n* = 100 randomly selected regions) versus those targeted by ZFP57 (darker colored). Known maternally methylated ICRs are shown as hatched boxes for comparison. ICM, inner cell mass. ns, not significant, *P < 0.05, **P < 0.01, ***P < 0.001; Mann–Whitney *U* Test

**Additional file 2.** Additional tables.

## Data Availability

Raw data for ChIP-seq and RNA-seq have been submitted to GEO under the accession number GSE123942.
